# FedEHR: A Federated Learning Approach towards the Prediction of Heart Diseases in IoT-Based Electronic Health Records

**DOI:** 10.3390/diagnostics13203166

**Published:** 2023-10-10

**Authors:** Sujit Bebortta, Subhranshu Sekhar Tripathy, Shakila Basheer, Chiranji Lal Chowdhary

**Affiliations:** 1 Department of Computer Science, Ravenshaw University, Cuttack 753003, India; sujitbebortta.cs@ravenshawuniversity.ac.in; 2 School of Computer Engineering, KIIT Deemed to be University, Bhubaneswar 751024, India; 3 Department of Information Systems, College of computer and Information Science, Princess Nourah bint Abdulrahman University, P.O. Box 84428, Riyadh 11671, Saudi Arabia; sbbasheer@pnu.edu.sa; 4 School of Computer Science Engineering and Information Systems, Vellore Institute of Technology, Vellore 632014, India

**Keywords:** machine learning, federated learning, Internet of Things, heart disease prediction, Electronic Health Records

## Abstract

In contemporary healthcare, the prediction and identification of cardiac diseases is crucial. By leveraging the capabilities of Internet of Things (IoT)-enabled devices and Electronic Health Records (EHRs), the healthcare sector can largely benefit to improve patient outcomes by increasing the accuracy of disease prediction. However, protecting data privacy is essential to promote participation and adhere to rules. The suggested methodology combines EHRs with IoT-generated health data to predict heart disease. For its capacity to manage high-dimensional data and choose pertinent features, a soft-margin L1-regularised Support Vector Machine (sSVM) classifier is used. The large-scale sSVM problem is successfully solved using the cluster primal–dual splitting algorithm, which improves computational complexity and scalability. The integration of federated learning provides a cooperative predictive analytics methodology that upholds data privacy. The use of a federated learning framework in this study, with a focus on peer-to-peer applications, is crucial for enabling collaborative predictive modeling while protecting the confidentiality of each participant’s private medical information.

## 1. Introduction

Research in the field of healthcare is crucial because of the significant impact it may have on people’s daily lives, as well as on the standard of living in their communities. Healthcare’s overarching mission is to improve people’s health and quality of life by reducing the prevalence of illness and injury. The IoT allows everyday things to connect to the internet and share data and processing power. The Internet of Things has the potential to aid in the creation of flexible and efficient applications in many fields, such as healthcare, environmental monitoring, and industrial control systems. The Internet of Medical Things (IoMT) is a new study area spawned by the IoT’s application in healthcare settings. Healthcare services, quality of life, and access to cost-effective solutions can all benefit from IoMT-based solutions integrated into the medical field [1]. Human biological information is collected in various formats, including health data, photographs, physical signals, etc. With today’s technology, researchers have easy access to data on a massive scale in biomedicine [2].

Multiple illnesses can now be detected, and warnings can be issued, thanks to wearable sensing devices such as smartwatches, wristbands, and many more. Wearable technology is on the rise, which is good news for efficient data collecting and the early diagnosis of diseases. Healthcare is a system that is formed to prevent, diagnose, and treat various health-related problems in humans. The broad implementation of Electronic Health Records (EHRs) has changed medical information storage, access, and analysis. EHRs store patient medical histories, treatment plans, test results, clinician notes, and other vital healthcare data. This plethora of data offers a unique opportunity to use modern data analysis for disease prediction and prevention. EHR incorporation into healthcare systems has improved the treatment of patients and enabled more proactive and individualised healthcare management. Electronic health records for disease prediction constitute a paradigm change in healthcare delivery. EHRs contain a wealth of patient data that clinicians can use to understand a patient’s medical history, lifestyle, genetics, and therapy reactions. This multidimensional approach allows healthcare providers to create predictive models that assess illness vulnerability, enabling early intervention and individualised prevention. The potential of EHRs for disease prediction is excellent, but data privacy, security, and ethics must be considered.

As healthcare-related technologies evolve, more and more information is becoming accessible from a wide range of sources. One of the problematic aims of contemporary civilisation is establishing a reliable healthcare infrastructure system [3]. Digitisation and data-driven decision-making have transformed healthcare in recent years. Data-driven approaches have always struggled due to a lack of domain-specific representative data; if ANNs are trained over data that are not statistically informative with respect to the problem being addressed, then they might not successfully extrapolate when applied in a real-world scenario, despite displaying satisfying behavior over training data.

**Figure 1:**
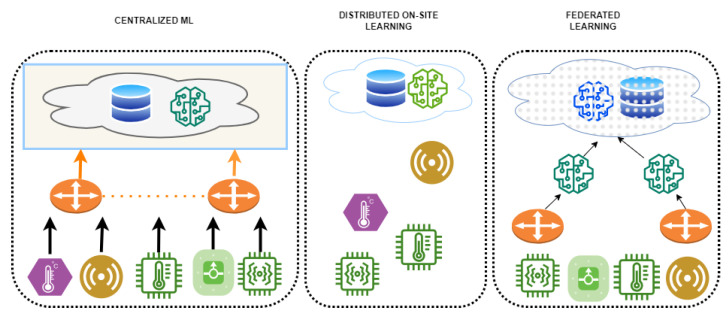
Comparison of different learning architectures.

The good news is that most of these issues may be solved by adopting federated learning (FL) [4], an effective method. In order to learn a model with the exchanging of data, FL is a solution for distributed machine learning (ML) challenges that is both privacy-preserving and GDPR-compliant. It can provide an approach for dealing with the security, confidentiality, and data governance constraints that prevent the sharing of medical data among various healthcare organisations by allowing the utilisation of the enormous quantity and wide range of healthcare data accessible from different sources, increasing the statistical power and generalizability of ML models. Several therapeutic areas, including imaging diagnostics, drug research, and genetics, have successfully employed FL [5]. Our study recommends a strengthened federated learning strategy based on a modified particle swarm optimisation method to address these issues. The approach seeks to improve precision and speed up convergence without compromising patients’ privacy. Such an approach has the potential to dramatically enhance healthcare outcomes and add to the efforts that are currently underway towards better healthcare delivery. Figure 1 provides the comparison of different learning techniques, viz., centralised machine learning, distributed on-site learning, and FL techniques.

The medical sector benefits significantly from the combination of IoT and FL. Wearable sensors, glucose monitors, and blood pressure cuffs are just a few examples of medical gadgets that can contribute to the real-time monitoring of patients’ health thanks to the Internet of Things. However, there are issues concerning patient privacy and data security associated with the usage of this data. By allowing numerous gadgets to work together on a model without sharing raw data, FL can alleviate these worries. This approach protects patients’ confidentiality while letting doctors analyse the data for patterns and trends. Furthermore, by spreading the burden across numerous devices, FL can assist in easing the computational and storage limits of IoT devices [6,7].

Despite the promise it holds, FL and IoT provide several difficulties, such as the following:

- Device heterogeneity: Internet of Things gadgets come in many types and forms, with a wide range of equipment specs and software options. Due to this diversity, creating a Federated Learning model compatible with a wide range of hardware may be challenging.
- Data Privacy and Security: FL introduces several privacy and security concerns while working with private data. Robust security protocols are essential for Federated Learning to guarantee the confidentiality of student information at all times.

Federated learning differs from traditional machine learning in various aspects, such as data center dispersal. The two methods aim at meeting learning objectives, but federated algorithms must consider the unstable networks and restricted upload capacity of edge devices. Reducing the overhead associated with communication is crucial in federated learning, as it is more prevalent than determining the overhead [8]. The communication overhead in federated learning can be assessed by counting the total number of cycles among the main server and subscribers. The effectiveness of the federated learning model may be assessed by assessing the precision of the classification after a set number of communication rounds [9]. Meta-heuristics may effectively solve FL difficulties such as resolution velocity, resilience, and the effectiveness of communication. This strategy reduces the optimal function of FL algorithms while preserving data privacy and increasing computing performance. Meta-heuristics are strategies employed to address difficult optimisation issues. These strategies significantly enhance FL algorithms’ efficiency [10].

Numerous novel meta-heuristic strategies have been proposed in recent research to reduce the fitness function in optimisation issues. Among these methods are the Slime Mold Algorithm (SMA) [11], the Harris Hawks Optimisation (HHO) [12], the Heap-Based Optimiser (HBO) [13], and the Sine–Cosine Algorithm (SCA) [14]. These methods work together to boost the rate of weight adaptation, fine-tune the neighbourhood searching procedure, perfect the computation of the dynamic fitness function, sidestep locally optimal solutions, quicken the convergence rate, and locate the best possible local solution.

Our distributed federated learning strategy enables on-device training and aids in training machine learning models on IoT networks. We present a heuristic by exploiting the Round-Robin algorithm to reduce the transmission of information costs among the central server and devices with limited resources. The goal of this work is to create a distributed, or federated, system that can predict how many people with heart illnesses will need to be hospitalised within a given year. Their extensively recorded medical histories, found in their Electronic Health Records (EHRs), serve as the foundation for this predictive research. These patients’ medical records may be stored on their smartphones or in the EHR platforms used by multiple hospitals. Regardless of the storage method, interdisciplinary work involving many stakeholders (agents) is necessary to develop a thorough hospitalisation prediction model on a worldwide scale. The main goal is to conceptualise the problem as a binary supervised classification problem. After that, a distributed approach based on soft-margin l1-regularised (sparse) Support Vector Machines (sSVM) will be created.

Support Vector Machines (SVMs) are the foundation of our chosen strategy because they have been shown to be good classifiers [14] and are capable of making reliable hospitalisation predictions [3]. The addition of sparse classifiers, a characteristic that emphasises the identification of a specific set of predictive attributes and encourages the elaboration of prediction justifications, greatly strengthens this approach [15]. We conducted a thorough review that took into account important criteria, such as communication volumes, accuracy, and transmission costs, in an effort to confirm the effectiveness of our suggested approach. In order to put our findings into context, we compared our approach to well-known benchmark standards like GA-SVM, SVM, and Fed-SVM. The results of this thorough analysis support the effectiveness of our suggested algorithm. Our method showed amazing efficiency in terms of communication volumes, exploiting inter-agent collaboration effectively while minimising the amount of data transferred. Furthermore, our strategy consistently outperformed the benchmark alternatives across a range of scenarios when evaluated for accuracy.

### Contributions

The proposed method must enhance model trustworthiness in Federated Learning (FL) systems where clients store their data locally. Our technique can enhance the dependability of centralised learning by updating the global model to be housed on IOT devices once all clients transmit their data to a central server. The main contributions of this work can be summarised as follows:

- We present the cPDS federated optimisation framework, a new framework for federated optimisation aimed at overcoming the sparse Support Vector Machine (SVM) conundrum. This plan stands out for its scalability, a key quality important in adjusting to the expanding healthcare data landscape. A key strength of cPDS is its ability to avoid the requirement for raw data exchanges, which is crucial in the healthcare industry where data privacy is of utmost importance. Notably, our research reveals that cPDS has a higher convergence rate than other centralised and distributed alternatives, along with a more favourable communication cost.
- In order to analyse a large dataset made up of de-identified Electronic Heart Records coming from the prestigious Boston Medical Centre, our ground-breaking methodology is rigorously applied. This database includes a wide range of people suffering from conditions related to the heart. Each patient’s profile is carefully crafted using a variety of relevant details, such as clinically significant aspects of their medical history, diagnostic insights, past hospital admissions, and demographic information.
- We explore the area of predicting patient hospitalisation scenarios within a predetermined time frame—specifically, a target year—by utilising the strong capabilities of the cPDS architecture. Our research leads to a fine distinction between patients who are thought to be potential candidates for hospitalisation and those who are thought to be unlikely to encounter such a result. We next carefully explain the experimental results and implications resulting from our predictive models and discuss them.
- The cPDS framework’s conceptual foundation extends beyond the unique context of our current study. Instead, it is applicable to a wide range of learning issues that share a composite “non-smooth + non-smooth” loss function objective. This pervasiveness resonates in the field of machine learning, where the goal is to minimise the functions highlighted by irregular regularisers. Similar to this, the distributed model predictive control setting, where the goal is to solve issues with similar characteristics, finds application for the cPDS framework.

The organisation of the remaining sections of this paper is as follows. Section 2 of this study will examine the history and relevant literature of federated learning and present examples of federated learning from earlier research. Section 3 outlines the evolution of ML and the traditional PSO algorithm. Section 4 details the proposed federated improved optimisation by examining the results is Section 5 of our suggested method, and Section 6 provides examples of how healthcare is improved as a result of FedEHR integration. To sum up the paper, Section 7 is the final section.

## 2. Related Work

The critical advantage of federated learning is that it allows researchers and AI developers to tap into a potentially vast pool of real-world data [15]. Instead of relying simply on the limited amount of publicly available datasets, this method [16] allows resources to be directed toward satisfying clinical objectives and addressing related technical challenges. Nevertheless, further study is required to determine the best algorithmic approaches for federated training, such as how to efficiently combine models or updates and make the system resilient to changes in the population. Because FL-based development assumes that the researcher or AI developer cannot review or observe every piece of data used to train the model, examining particular instances of failure and explaining why the current model performs poorly on them is challenging. FL has been successful in several contexts recently, regardless of these drawbacks. Centralised, decentralised, and federated learning are just a few of the machine learning deployment strategies described by the researchers in reference [17]. They have explained the evolution of structures for machine learning in detail, showing a lot of thought and effort. In reference [18], the researchers of a study on patients with conditions probable to necessitate hospitalisation developed a federated-learning-based model. They used information gathered from dispersed electronic health records (EHRs). The authors presented the clustering-based strategy for dual splitting to employ FL to address the problem of large-scale sparse computing. The precision of the classifier predictions was comparable when using their approach—the authors of reference [19] evaluated and tested the three FL-based techniques using the MNIST dataset. We also used a *t*-test with a Bayesian correlation. FedAvg outperformed CO-OP and FSVRG in their evaluation when the maximum number of client uploads was set to 10,000. Each client receives the same amount of data thanks to their implementation of equal information distribution.

To enhance the method’s precision and convergence speed, the authors of reference [20] proposed a tweaked variant of the traditional FL. They developed the FedMA method, a layer-wise adaptation of the FL algorithm, to apply Bayesian non-parameterised techniques to heterogeneous data. In convergence, accuracy, and message size reduction tests, their proposed FedMA came out on top. In reference [21], the authors investigated the aspects that affect data privacy in a distributed implementation environment for FL algorithms, including technological problems. Several different optimisation methodologies for FL deployment were examined, and their characteristics and outcomes were discussed in the paper. They have also examined the potential financial effects of federated learning. An approach for weighting classes depending on their contributions to local models is proposed in reference [22]. Machine-learning-based methods can aid in detecting COVID-19 using data from patients’ chest X-rays. In reference [23], a FL variant was proposed to find COVID-19 with more accurate predictions than traditional machine learning methods.

In reference [24], the researchers suggest a blockchain-based, federated-learning solution to the data confidentiality issue in IoMT-based healthcare systems. They proposed a blockchain-based, hybrid solution to the problem of confidentiality of user data that relied on federated learning and the maximum approximation of the Gaussian mixture model. Their method illustrates how IoMT data training may be accomplished with local privacy to prevent leakage. A distributed, federated learning architecture that takes security into account was proposed as a means of predicting heart disease. Health data confidentiality and heart disease forecasting were both improved by FedMA and M-ABC. This resulted in improved cardiac diagnosis, education, and coordination. Baseline federated learning FedAvg, FedMA, and FedMA with the PSO optimiser techniques were used to evaluate the structure, and the parameters and efficacy of the interaction based on model predictions were analysed. The system provides enhanced performance metrics, including classification error, accuracy, sensitivity, and sensitivity to noise. The design achieves its highest level of accuracy in 12% fewer rounds, while increasing precision by 1.8%, sensitivity by 7.1%, and classification error by 7%. IoMT scalability was affected by the model’s learning rate at client sites. Privacy-conscious healthcare forecasting benefited from the use of alternative feature selection and optimisation methodologies [25]. In their paper, “Federated Learning for Healthcare Informatics”, Patel et al. [26] investigate how Federated Learning might be used more effectively, comprehending and employing medical data from various resources to enhance patient care or results. The researchers emphasise the issues associated with information variation, confidentiality, and adherence to regulations while discussing the applications of Federated Learning in healthcare, such as disease detection, drug development, and tailored treatment. They offer potential avenues to further study and solutions to existing problems. To optimise the convergence speed of federated learning, the researchers in reference [27] proposed a fast-convergent strategy that automatically selects all devices at every round of training the framework. Their approach uses a precise and effective computation to transmit a nearly optimal distribution of device selection, increasing the convergence rate. In order to improve the effectiveness and safety of collaborative training in a federated setting, the authors of reference [28] suggest a new method that uses Secret Sharing (SS). The recommended approach is superior to the dishonest paradigm because malicious actors can stray from the protocol. In addition, the proposed method yields exceptional results in terms of both execution time and communication cost for the simulations that are examined.

In reference [29], authors offer a system design for scalable Federated Learning with the goal of facilitating confidential and secure cooperation across many parties. In order to overcome the difficulties of Federated Learning, such as flexibility, disparity, and anonymity, the researchers suggest a framework for systems which employs safe aggregation, differential privacy, and adaptive optimisation. Through addressing the under-representation of instances of positives in standard mammography imaging datasets, Amelia and Tardy [30] aim to boost the performance of CAD systems for breast cancer detection. To improve federated learning, this research presents a new memory-aware curriculum learning method in which local models compute their local data to update the global model [31,32]. The global model training examples will be ordered according to the proposed curriculum. The method is used with unsupervised domain adaptation to handle domain switching without compromising data confidentiality. The proposed method is tested on three commercial clinical datasets, with results showing a 5% and 6% improvement in ROC-AUC and PR-AUC, respectively, over a standard federated setup.

## 3. Proposed Framework

By seamlessly merging a cutting-edge soft-margin L1-regularised sparse Support Vector Machine (sSVM) classifier with a ground-breaking iterative Cluster Primal Dual Splitting (cPDS) method, the proposed framework offers a comprehensive solution for a federated heart disease detection system. With a three-tiered architectural model that includes healthcare sensors and wearables at the foundational layer, communication channels at the intermediary layer, and culminating in the uppermost stratum hosting cloud servers and intelligent machine-learning-based analytics for data interpretation, this convergence of advanced techniques is designed to address the complexities of large-scale sSVM problems.

The framework’s main component is a soft-margin L1-regularised sparse Support Vector Machine (sSVM) classifier, which was specifically developed to tap into the potential of massive amounts of medical data for the precise prediction and diagnosis of cardiac disorders. The sparse design of this classifier gives it the capacity to emphasise only the most important features, streamlining interpretability. It is adept at handling complicated data patterns and variable relationships that are inherent in heart health data.

In addition, the developed Cluster Primal Dual Splitting (cPDS) algorithm emerges as a significant enabler, crucial in overcoming the difficulties brought on by the healthcare data landscape’s intrinsic decentralisation. This iterative technique combines primal and dual characteristics to ensure accurate and quick convergence, making it a reliable mechanism for resolving the challenging optimisation issues inherent to large-scale sSVM. The decentralised network’s outcomes are optimised thanks to the judicious use of matrices like “j” and “j”, which boost the cPDS algorithm’s effectiveness.

The intermediary layer ensures data integrity and privacy, paramount considerations within the healthcare domain, paving the way for seamless data transfer to the upper tiers. The intermediary layer is dedicated to the establishment of communication channels, facilitating the secure and swift exchange of data between the myriad healthcare sensors and wearables dispersed across diverse locations.

The topmost layer of the architectural hierarchy is where cloud servers are highlighted. These servers employ cutting-edge machine-learning-based algorithms that mine the compiled data in depth for information that helps physicians make well-informed decisions. The use of complicated algorithms is made possible by the cloud-based architecture, which also ensures scalability and accessibility without placing pressure on nearby devices.

**Figure 2:**
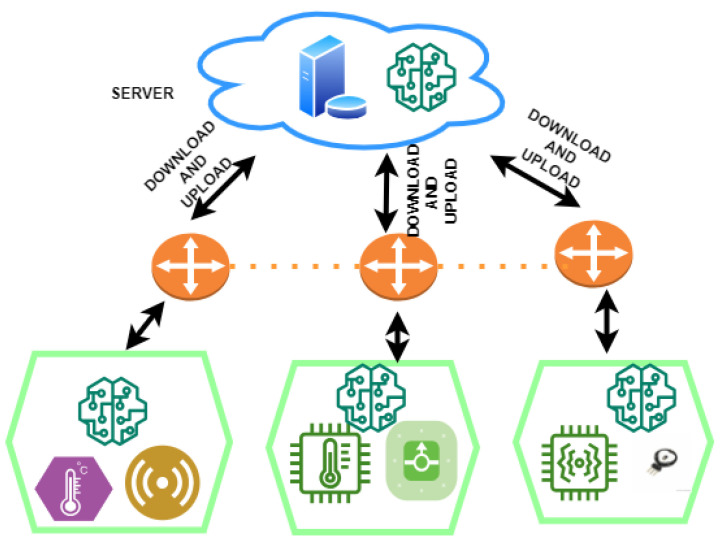
Overview of the architecture for proposed federated framework.

The proposed framework combines cutting-edge techniques to create a federated system for diagnosing heart disease. These methodologies take the shape of soft-margin L1-regularised sSVM classifiers and cPDS algorithms. Incorporating healthcare sensors, communication channels, and cloud servers, this system operates within a meticulously planned architectural framework, creating a unified ecosystem that can revolutionise heart disease diagnosis through accurate and decentralised data-driven analytics. Figure 2 provides the overview of the proposed framework.

## 4. Problem Formulation

There is a growing need for effective computational models that can draw insightful conclusions from this enormous information reservoir as the volume, variety, velocity, and veracity (the four V’s) of clinical data continue to increase. The use of these computational tools has the potential to improve patient care by helping to create efficient healthcare systems, detecting the root causes of diseases, providing individualised and cost-effective medical interventions, and more. Our inspiration comes from the complex challenges that frequently arise in the medical field and that can be formulated as binary supervised classification problems that may be solved by using Support Vector Machines (SVMs). These applications cover a wide range of scenarios, including the automated detection of obstructive sleep apnea syndrome [6], forecasting hospitalisations due to cardiac events [3], projecting medication adherence patterns among heart failure patients [4], and diagnosing cancer. The body of literature now in existence emphasises the effectiveness of sparse classifiers, which are distinguished by their reliance on a constrained number of discriminative features. Beyond the training dataset, these classifiers exhibit strong predictive ability and favourable generalisation performance [7,8]. Surprisingly, both in terms of the model’s inherent structure and the interpretive significance of the produced results, these models simultaneously provide a higher level of interpretability. The ability to comprehend results is a quality that is of utmost relevance in promoting healthcare professionals’ confidence in algorithmic method results. The privacy of patient data, however, is a salient problem in the medical field, which has sparked recent research initiatives [9,10,11]. De-identified data are prone to privacy breaches, making people exposed to identification, as demonstrated by the Netflix Prize and the Massachusetts Group Insurance Commission (GIC) medical information repository. These incidents highlight the potential for sensitive data to be compromised once a centralised entity has access to and is processing large databases. In the context of the Precision Medicine Initiative [12], where the future incorporation of people’s genomic data emphasises the necessity of maintaining data sensitivity, this vulnerability assumes further significance.

Three key issues that are intricately related to healthcare data management are the focus of our work: (1) the spatial dispersion of data across various sources, including hospitals, medical offices, home monitoring devices, and patient smartphones; (2) the growing influx of healthcare data that calls for scalable frameworks to handle the data deluge; and (3) the impracticability or unfavorability of centralising data in a single repository. In particular, the traditional strategy of centralising all data makes it possible to implement privacy preservation strategies like k-anonymity [13]. But this centralisation creates a single point of weakness that can be breached, exposing personally identifiable information about a large number of people. Additionally, creating a centralised data repository necessitates significant infrastructure investments and navigating complex information governance requirements, including obtaining rights for data processing and storage. As a promising alternative, a novel decentralised computational paradigm, that is based on treating the accessible data as members of a federated virtual database, emerges. This method avoids the traditional methods of centralised data collecting, processing, and raw data exchange, perhaps providing a solution to the problems outlined above.

We look at a dataset obtained from an Electronic Health Record (EHR) system that contains crucial patient demographic information including age, gender, and race. The additional relevant physical characteristics included in this dataset include height, weight, and Body Mass Index (BMI). The patients’ medical histories are scrupulously recorded, including diagnosis, treatments, trips to the doctor’s office, and a detailed list of all medications administered. The collection represents patient profiles ranging from *i* = 1 to n because each patient is identified by a unique feature vector. Our main goal is to predict whether a certain patient would require hospitalisation within a certain time frame, such as the year after the records review.

(1) $$\min_{\theta ,\theta_{0}}\sum_{i=1}^{n}k_{i}\left(\theta ,\theta_{0}\right)+0.5\psi {∥\theta ∥}_{2}^{2}+\rho {∥\theta ∥}_{1}$$

where $\left(\theta ,\theta_{0}\right)$ denotes two vectors belonging to the kernel function $k_{i}\left(\theta ,\theta_{0}\right)$ such that, $\theta \in R^{d}$, $\theta_{0}\in R$ represent the hyperplane $k_{i}\left(\theta ,\theta_{0}\right)=\max\left\{0,1-\ell_{i}\left(\varphi_{i}^{\tau }\theta +\theta_{0}\right)\right\}$ indicates a hinged loss function pertaining to sample *i*, with ψ and ρ depicting the co-efficients for penalty, and the l1-norm term ${∥\theta ∥}_{1}$ represents sparsity.

Thus, the sSVM problem defined in this article finds the classifier $\left(\theta ,\theta_{0}\right)$ by solving for the following problem:

(2) $$\begin{array}{c} \min_{\theta ,\theta_{0}}0.5{∥\theta ∥}^{2}+c{\sum_{i=1}^{n}\zeta_{i}+\varepsilon ∥\theta ∥}_{1}\\ s.t.,\zeta_{i}\geq 0,\forall i,\\ \ell_{i}\left(\varphi_{i}^{\tau }\theta +\theta_{0}\right)\geq 1-\zeta_{i},\forall i. \end{array}$$

Further, in a decentralised setting comprising *m* agents, we can rewrite the above Equation (2) as below:

(3) $$\begin{array}{c} \min_{\theta ,\theta_{0}}\sum_{j=1}^{m}\left\{\sum_{i=1}^{n_{j}}\left[1-x_{ji}\right]+0.5\psi_{j}{∥\theta_{j}∥}_{2}^{2}+\rho_{j}{∥\theta_{j}∥}_{1}\right\}\\ s.t.,\gamma_{ji}\left(\ell_{ji}\left(\varphi_{ji}^{\tau }\theta_{j}+\theta_{j0}\right)-x_{ji}\right)=0,\forall j,i;\\ \theta_{1}=\theta_{2}=\cdots =\theta_{m};\\ \theta_{1,0}=\theta_{2,0}=\cdots =\theta_{m,0}, \end{array}$$

where each agent in the framework represented as parameter *j* holds $n_{j}$ samples, such that, $n=\sum_{j=1}^{m}n_{j}$, and $x_{ji}=\ell_{ji}\left(\varphi_{ji}^{\tau }\theta_{j}+\theta_{j0}\right)$.

### 4.1. Cluster Primal–Dual Splitting Problem

Then, using Equation (1) as an example, we offer a broad-spectrum decentralised primal–dual splitting strategy that has been carefully designed to address optimisation issues characterised by the “nonsmooth + nonsmooth” features.

Let us look at a network of agents who are each given access to a portion of the data and who are working together to solve the optimisation problem shown in (1) by utilising the complete set of data. The two possible outcomes of this project are as follows: first, each agent has a collection of various samples, creating a semi-centralised framework; second, each person keeps their own personal data, frequently kept on devices like smartphones, creating a fully-decentralised paradigm. In our healthcare environment, the first case corresponds to hospitals processing patient data inside their respective areas of responsibility, with interhospital communication permitting collaborative Equation (1) problem-solving. As an alternative, in the latter case, patients and their counterparts actively exchange data while working together to solve the same problem (1). A hybrid scenario that combines aspects of the semi-centralised and fully-decentralised strategies is also a possibility. Regardless of the scenario, the group of agents designated as “m” is linked together by a communication network that is aptly depicted in an undirected graph G=(V,E), where *V* contains the vertex set 1,2,…,m, and *E* stands for the edge set. We uphold two fundamental presumptions throughout this discussion: first, that the graph *G* remains linked; and second, that information exchange inside the graph only takes place between nearby agents.

Thus, following the Metropolis rule, we have the doubly stochastic matrix as,

(4) $$\omega_{ij}=\left\{\begin{array}{c} \frac{1}{\max\left\{\deg\left(i\right),\deg\left(j\right)\right\}+1},if\left(i,j\right)\in E\\ 0,if\left(i,j\right)\notin Eandj\neq i\\ 1-\sum_{h}\omega_{ih},ifi=j \end{array}\right.$$

Then, we present our general decentralised primal–dual splitting approach, meticulously developed to address optimisation problems characterised by the “nonsmooth + nonsmooth” paradigm, as illustrated by Equation (5) below:

(5) $$\begin{array}{c} \min_{\left\{x_{j},y_{j}\right\},\forall j}\sum_{j=1}^{m}\left\{G_{j}\left(x_{j}\right)+F_{j}\left(y_{j}\right)\right\}\\ s.t.,\Gamma_{j}\left(A_{j}x_{j}-y_{j}\right)=0,\forall j,\\ x_{1}=x_{2}=\cdots =x_{m}. \end{array}$$

### 4.2. Heuristic for Transmission Cost Reduction

Within the context of federated learning, the Round-Robin algorithm is an established technique that can be strategically used as a heuristic to reduce transmission costs while assuring fair server use. By successively choosing participant nodes for model updates, Round-Robin carefully distributes communication resources in this situation, preventing uneven traffic distribution and potentially congested networks. This method streamlines data transfer between federated learning nodes, maximising bandwidth usage and reducing the cost associated with excessive data transfer as a result. Therefore, the Round-Robin algorithm emerges as an effective way to lower transmission costs by virtue of its judicious load allocation, enabling the effective orchestration of collaborative machine learning tasks over a distributed network of federated computers.

The pseudo code presented in Algorithm 1 cycles through the participant nodes and chooses a subset that will send data and model changes to the server in a fair and timely way. This method effectively uses the network resources that are available while preventing any single node from being inundated with communication responsibilities. It promotes fairness and minimises the transmission costs associated with network congestion and uneven workloads by routinely rotating the chosen node, preventing any particular participant node from being preferred over others.
**Algorithm 1** Round-Robin-algorithm-based traffic distribution**Require:** $V,S_{agg}$1:V← set of vertices or participant nodes.2:$S_{agg}\leftarrow$ set of server or aggregator nodes for federated learning.3:**Initialize:** pointer variable to current node v∈V4:Check for *m* agents and penalty coefficients psi and rho5:**for**$S_{agg}$ in ready state **do**6:    Find $NEXT\left[V\right]$7:    Set $v=NEXT\left[V\right]$8:    **if** $NEXT\left[V\right]=NULL$ **then**9:        Set $NEXT\left[V\right]=v\left[0\right]$ and execute cyclically10:    **end if**11:    Compute $v\to S_{agg}$12:**end for**

## 5. Results

### 5.1. Experimental Analysis

For our investigation, we used the MATLAB R2020a programming environment for incorporating the proposed framework, which was run on a device with an Intel^®^ CoreTM i7 processor running at 4 GHz and 16 GB of RAM. This computational setup’s goal was to run a lengthy simulation with 1000 communication rounds that served as a thorough assessment of the performance displayed by our suggested framework.

We employed typical Federated Learning (FL) techniques at the client level, with a clear emphasis on the implementation of Support Vector Machine (SVM) classifiers, to benchmark the effectiveness of our system. Because of this, the range of traditional algorithms we used includes, notably, GA-SVM, SVM, and Fed-SVM. We chose the GA-SVM and Fed-SVM algorithms to depict the improvements in the basic SVM model by adding a genetic algorithm (GA) and FL approach with SVM, which resulted in instantiations known as GA-SVM and Fed-SVM.

The efficiency of the suggested framework in the context of heart disease prognosis and diagnosis is evaluated comprehensively by our inquiry. The results of this evaluation procedure are directly compared to well-known state-of-the-art methods, such as GA-SVM, SVM, and the Fed-SVM techniques. The metrics for comparison cover a broad range of criteria, including prediction accuracy, the total utility, communication effectiveness, and the innate impact of localised epochs on the obtained accuracy.

In designing our experimental configuration, we decide on a setup that consists of five client nodes that are designated as Healthcare Service Provider (HSP) clients, in addition to one HSP server node. Our evaluations and comparisons are built on top of the selected setup. However, it is important to emphasise that the suggested framework is scalable, giving it the capacity to support a wider range of HSP client nodes if that is deemed appropriate and essential.

### 5.2. Discussions

The concluding component of our research focuses on the presentation, analysis, and interpretation of the findings from the thorough assessments we conducted of our proposed federated heart disease diagnosis system. This part serves as a platform for revealing the empirical results, shedding light on the framework’s efficacy and prowess through thorough experimentation and comparison with cutting-edge methodologies. The discussion that follows combines these empirical findings with essential theoretical understandings, allowing for a thorough understanding of the implications and importance that our framework has in the context of heart disease prognosis and diagnosis. The results are presented in a way that not only offers quantitative evaluations of various performance measures, but also qualitative viewpoints that help us understand the strengths and weaknesses of the framework as a whole.

**Figure 3:**
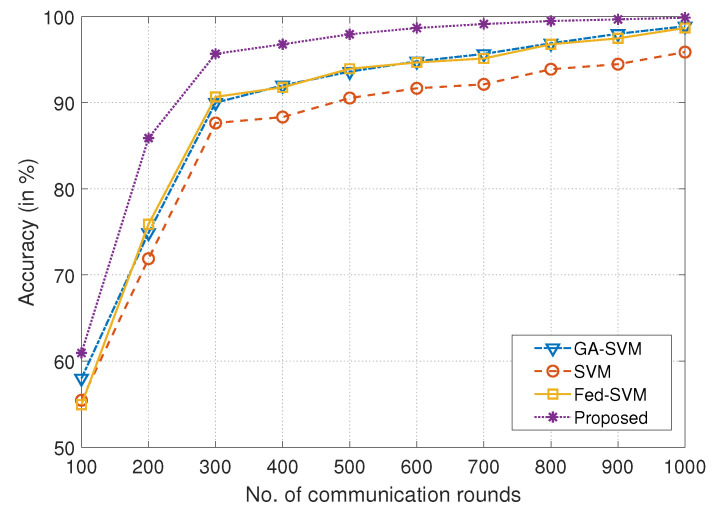
Comparison of number of communication rounds in the federated architecture with accuracy achieved for proposed framework and benchmark algorithms like GA-SVM, SVM, and Fed-SVM.

As a visual depiction of an extensive comparison analysis, the plot that is being given in Figure 3 tracks the development of four different algorithms throughout a range of communication rounds on the *x*-axis and prediction accuracy on the *y*-axis. We rigorously evaluate and compare these algorithms, GA-SVM, SVM, Fed-SVM, and our proposed Federated SVM with soft margin L1-regularisation, with the overall goal of highlighting their individual performance trajectories. The algorithms’ prediction accuracy develops on a dynamic trajectory as the communication rounds move down the *x*-axis, revealing a complex interplay between iterations and predictive accuracy. A characteristic curve that represents the GA-SVM algorithm’s performance, an example of advanced optimisation, shows how its accuracy changes over time as communication increases. The SVM method, which represents a conventional technique, follows a different path that is distinguished by a subtly different accuracy progression. In Table 1, a detailed statistical summary, depicting the comparison of accuracy obtained by different models corresponding to different communication rounds, is presented.

In addition to this, the accuracy trajectory of the Fed-SVM algorithm is shown, evoking the principles of federated learning. Its unique trajectory depicts the overall effect of distributed processing across nodes, resulting in distinctive patterns of accuracy growth. The trajectory of our suggested Federated SVM with soft margin L1-regularisation, however, stands out clearly from the rest. The curve clearly outperforms the benchmark algorithms since it shows a steadily increasing growth. The accuracy of our suggested architecture steadily improves as communication rounds add up, significantly outpacing the accuracy trajectories of GA-SVM, SVM, and Fed-SVM. This unmistakable visual illustration perfectly captures the spirit of our research project and highlights the ability of our suggested strategy to produce improved predictive accuracy in the field of heart disease prognosis.

**Figure 4:**
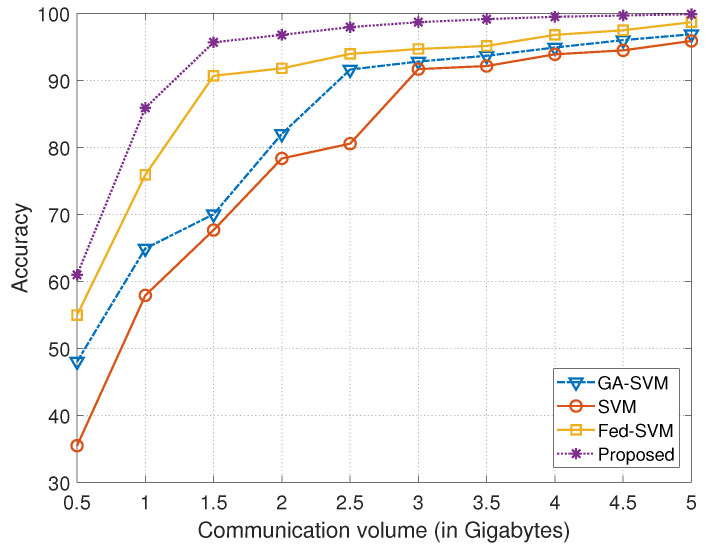
Comparison of communication volume in gigabytes with the achieved accuracy for GA-SVM, SVM, and Fed-SVM, along with proposed algorithm.

The graphical representation provided in Figure 4 is a useful illustration of a thorough comparative analysis, mapping the performance of four different algorithms over a range of communication volumes indicated on the *x*-axis in gigabytes against their corresponding prediction accuracy displayed on the *y*-axis. In order to reveal their unique performance dynamics in a real-world communication setting, the algorithms under consideration—GA-SVM, SVM, Fed-SVM, and our novel Federated SVM with soft margin L1-regularisation—have been meticulously examined and contrasted. Table 2 provides the statistical summary of the accuracy obtained using different models corresponding to different communication volumes (in gigabytes).

The growth of the algorithms’ accuracy trajectories is coupled with the progression of the communication volume along the *x*-axis, shown in gigabytes, demonstrating complex patterns of accuracy evolution under varied communication loads. The GA-SVM algorithm’s trajectory, which makes use of cutting-edge optimisation paradigms, is artfully depicted, showing how its distinctive accuracy trends change in response to changing communication volumes. At the same time, the SVM algorithm, which represents a conventional technique, takes a particular route and displays its own unique accuracy dynamics when communication volume varies. The trajectory of the Fed-SVM algorithm, which personifies the essence of federated learning, is equally compelling. This trajectory captures the effects of dispersed processing among network nodes, leading to a distinctive pattern of accuracy evolution impacted by shifting communication volumes.

But the trajectory of our suggested Federated SVM with soft margin L1-regularisation stands out, without a doubt, as the top performance. The curve on the plot, where the *x*-axis represents communication volume and the *y*-axis represents accuracy, regularly outperforms the benchmark algorithms. Our suggested system greatly outperforms GA-SVM, SVM, and Fed-SVM in terms of accuracy as communication volumes rise. This graphic representation underlines the major point of our study project and admirably illustrates the superior predictive accuracy and effectiveness of our creative strategy in the field of heart disease prediction.

**Figure 5:**
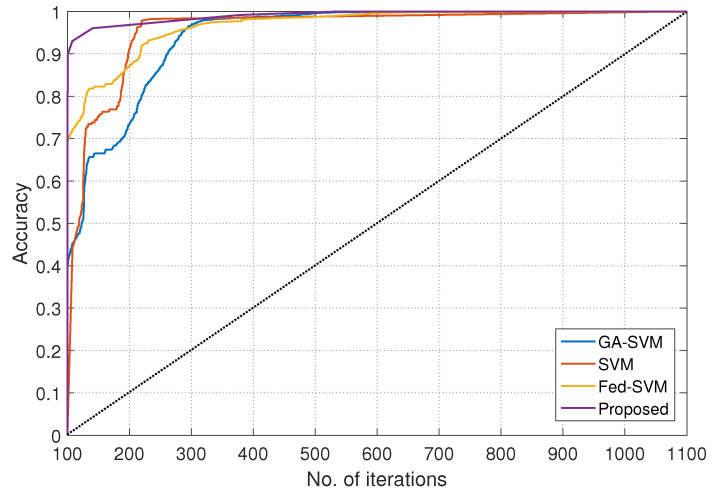
Number of iterations, along with accuracy, for GA-SVM, SVM, Fed-SVM, and proposed algorithm.

The resulting graphical representation in Figure 5, with the *x*-axis defining iteration numbers ranging from 100 to 1100, summarises a rigorous comparison investigation and provides insights into the performance of four different algorithms throughout a variety of epochs. The *y*-axis beautifully displays the matching accuracy levels attained using these techniques. These meticulously scrutinised algorithms—GA-SVM, SVM, Fed-SVM, and our ground-breaking Federated SVM with soft margin L1-regularisation—go through intense scrutiny to reveal their individual dynamics over a range of epochs, resulting in a thorough understanding of their predictive power.

The accuracy trajectories of these algorithms unfold with complex patterns of evolution as epoch numbers increase along the *x*-axis, from 100 to 1100, illustrating how they respond to changing epochs. The trajectory of the GA-SVM algorithm, which is characterised by sophisticated optimisation methods, is elegantly represented, providing insights into its various accuracy patterns as epochs develop. The SVM algorithm, a representative of traditional techniques, simultaneously plots its own unique course and communicates its accuracy dynamics with shifting epoch numbers. The Fed-SVM algorithm’s trajectory, which illustrates the concepts of federated learning, shows its unique path as it runs concurrently. This trajectory captures the overall effect of distributed processing across network nodes, resulting in a distinctive accuracy pattern that changes over time. However, what sticks out most is our suggested Federated SVM with soft margin L1-regularisation. This trajectory continuously climbs steeper and outperforms the benchmark algorithms. Our suggested framework constantly obtains improved accuracy levels as epoch numbers increase, outperforming the performance of GA-SVM, SVM, and Fed-SVM by a significant margin. This graphical representation effectively communicates the main idea of our research project, highlighting the higher accuracy and predictive capacity of our innovative strategy in the field of heart disease prediction.

**Figure 6:**
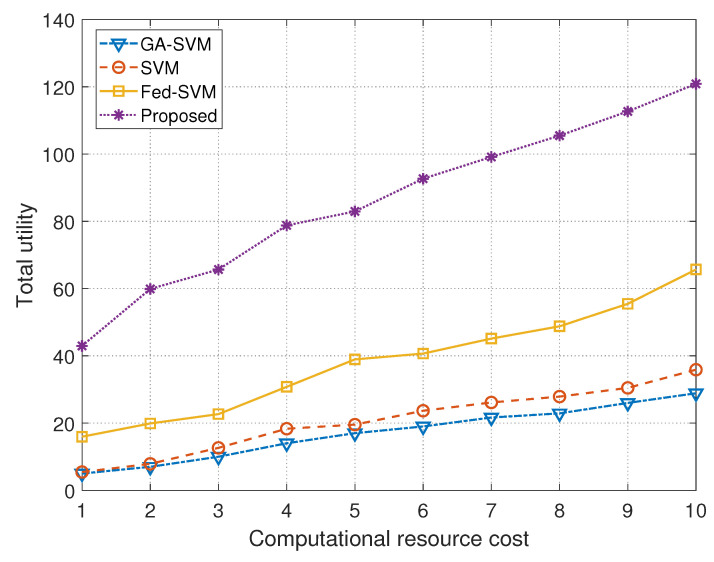
Plot comparing computational resource cost with total utility for GA-SVM, SVM, Fed-SVM, and proposed algorithm.

The next graphical depiction in Figure 6 encompasses a thorough comparative analysis and provides a comprehensive overview of the performance of four different algorithms when compared to the cost of computational resources. The plot’s *x*-axis painstakingly shows the cost of computational resources, and the *y*-axis thoughtfully shows the matching overall utility values attained using these algorithms. The algorithms under consideration—GA-SVM, SVM, Fed-SVM, and our ground-breaking Federated SVM with soft margin L1-regularisation—go through a thorough evaluation in this rigorous analysis to reveal their individual trajectories in terms of value in relation to computational resource expenditure. Table 3 presents a detailed statistical summary of total utility compared with computational resource cost.

The skillfully represented computational resource cost along the *x*-axis serves as a key parameter that establishes the framework for comprehending how these algorithms operate under various computational limitations. A clear image of the GA-SVM algorithm’s utility in relation to computing resource cost can be seen in its trajectory, which is characterised by advanced optimisation techniques. It shows a distinct pattern of performance that changes precisely as resource costs fluctuate. While computing resource costs fluctuate, the traditional SVM method follows a different route and reveals its own specific utility trends. The trajectory of the Fed-SVM algorithm, a representative of federated learning principles, unfolds its unique utility dynamics under various computational resource limitations, capturing the cumulative impact of decentralised processing across network nodes. But the trajectory of our suggested Federated SVM with soft margin L1-regularisation really stands out. This trajectory outperforms the benchmark algorithms with a consistently steeper climb. The overall utility of our suggested approach continuously outperforms that of GA-SVM, SVM, and Fed-SVM, even as the cost of computational resources varies. This graphical representation underlines the fundamental principle of our research project and demonstrates the greater usability and effectiveness of our unique strategy for the early detection of heart disease.

**Figure 7:**
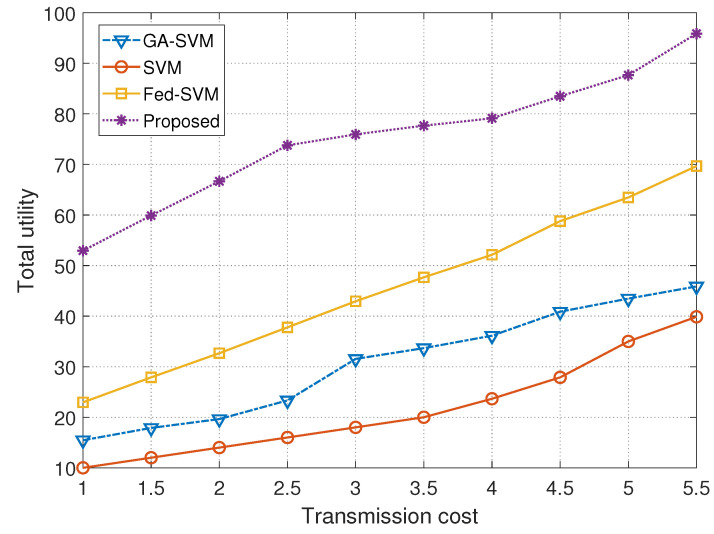
Comparison of transmission cost with total utility for GA-SVM, SVM, Fed-SVM, and proposed algorithm.

The graphical representation in Figure 7 offers a nuanced perspective on the performance of four different algorithms throughout a spectrum of transmission costs. It is a thorough embodiment of a rigorous comparison analysis. The plot’s *x*-axis precisely defines the transmission cost, and the *y*-axis deftly depicts the matching total utility values attained by these algorithms. The algorithms under consideration—GA-SVM, SVM, Fed-SVM, and our novel Federated SVM with soft margin L1-regularisation—undergo rigorous investigation in this complex evaluation, revealing their individual trajectories in terms of usefulness in conjunction with various transmission cost concerns. Table 4 provides a statistical summary of the total utility incurred by different models corresponding to transmission cost.

A crucial parameter that has been strategically positioned along the *x*-axis is the transmission cost, which lays the groundwork for understanding how these algorithms perform in various transmission cost scenarios. A clear picture of the GA-SVM algorithm’s utility in relation to transmission cost can be seen in its trajectory, which stands out for its sophisticated optimisation tactics. The trajectory shows a characteristic pattern of performance progression as transmission costs change. The SVM algorithm, a symbol of traditional methods, plots its own route in parallel and reveals its separate utility trends when transmission costs change. In addition, the Fed-SVM algorithm’s trajectory, which represents federated learning paradigms, captures the overall effect of distributed processing across network nodes and reveals the dynamics of its special usefulness in response to changing transmission costs.

The trajectory of our suggested Federated SVM with soft margin L1-regularisation, however, really stands out. This trajectory outperforms the benchmark algorithms by beginning a steadily steeper ascent. The overall utility of our suggested approach regularly outperforms that of GA-SVM, SVM, and Fed-SVM by a significant margin as transmission costs change. This graphical illustration supports the main finding of our study and demonstrates the increased usefulness and effectiveness of our ground-breaking method for anticipating heart disease.

## 6. Limitations and Challenges

It is important to recognise some limitations, even if the suggested methodology offers intriguing opportunities for improving the prediction and detection of cardiac illnesses through the combination of EHRs and IoT data. First, both the quality and quantity of the accessible data are crucial to the model’s efficacy. Obtaining complete and varied EHRs and IoT-generated health data might be difficult in practice, which could cause biases in the predictive model. It is still crucial to address data heterogeneity and make sure that representative datasets come from varied healthcare settings.

The proposed federated learning framework’s privacy-preserving feature is crucial for protecting patient data, but due to decentralised model updates among participant nodes, it may cause communication overhead and latency. Finding a compromise between effective model convergence and strong privacy protection is a difficult problem that requires more research. Additionally, when working with disproportionately massive data sets or a greater number of collaborating healthcare facilities, the cluster primal–dual splitting algorithm’s scalability for tackling large-scale sSVM problems may run into problems. Future research should focus on addressing these scaling issues and improving the algorithm’s performance in these situations.

## 7. Conclusions and Future Work

The development of a Health Service Provider (HSP) system capable of real-time patient data collecting has emerged as a crucial undertaking in the context of early disease identification and prompt treatment. Intelligent healthcare systems have the ability to quickly reduce health risks and save lives, particularly in distant situations when medical intervention is limited. Predicting survival outcomes in individuals with heart disorders remains one of many medical problems that is extremely difficult. A seamless flow of user data is hampered by the complexity of healthcare systems, which also extends to the delicate areas of privacy and security. Our study offers a ground-breaking hybrid federated learning approach to handle these complex issues, aiming to improve the accuracy of heart disease prediction while also resolving the privacy complexities present in healthcare systems.

The goal of this research project is to develop a federated learning model that can predict when patients with cardiac issues may need to be admitted to the hospital. Our coordinated efforts have resulted in the creation of the cluster Primal–Dual Splitting (cPDS) algorithm, a decentralised framework. This ground-breaking approach, designed to handle the sparse Support Vector Machine issue, produces classifiers that rely on a minimal number of features, improving the interpretability of the classification results. Notably, the cPDS algorithm sets itself apart from the numerous alternatives investigated during our work by virtue of an increased convergence rate, presenting it as a benchmark. This approach’s adaptability to a broad range of binary classification problems highlighted by distributed data is one of its most salient features.

Our proposed approach’s intrinsic adaptability to move through a range of operating settings, including both fully-centralised and fully-decentralised configurations, is a key benefit. The key to our strategy is to frame the main healthcare issue as a binary classification conundrum. The scope of information processing ranges from the level of individual patients—often facilitated by their personal devices like smartphones—to the upper echelons of healthcare institutions charged with the duty of maintaining patient data. The necessity to minimise two non-smooth terms characterises a wide range of issues, and the cPDS framework, acting as an all-encompassing paradigm, extends its potential reach to these problems. In order to demonstrate the effectiveness of our suggested approach, we conducted a thorough analysis that included three crucial performance indicators: communication volumes, accuracy, and transmission costs. In order to create a comprehensive benchmark, we compared our strategy to well-known alternatives including GA-SVM, SVM, and Fed-SVM. Our suggested strategy has resolutely shown its viability through this thorough study. Our programme skillfully grasped the concepts of decentralised learning while minimising the amount of data sent when conversation volumes were taken into account. Furthermore, our method continuously demonstrated superior prediction capabilities across a wide range of circumstances when evaluated for predictive accuracy.

Moreover, our method proved to be the pinnacle of computing efficiency in the field of transmission costs. It skillfully strikes a balance between pursuing optimal utility and reducing the computational load associated with data transmission inside the network. Together, these results demonstrate the enormous value of our suggested approach, placing it as a reliable option for anticipating heart-disease-related hospitalisations in the context of a distributed and collaborative environment.

The suggested approach provides a strong platform for future research in the field of predictive analytics for heart disorders. Future research should concentrate on improving data collecting methods, maybe by incorporating cutting-edge innovations like federated data sharing platforms and privacy-preserving data synthesis methods. By identifying complex patterns in healthcare data, it may be possible to further increase predictive accuracy by utilising advanced machine learning algorithms.

Furthermore, further research is needed to accept bigger and more varied datasets due to the scalability and effectiveness of federated learning algorithms in a healthcare environment. This includes creating peer-to-peer federated learning frameworks that are efficient and can manage highly dimensional data while maintaining data privacy. For federated learning systems to be ethically and legally compliant, it is crucial that healthcare organisations, data scientists, and regulatory agencies work together to build common guidelines. Therefore, to advance the state-of-the-art in cardiac disease prediction while adhering to the fundamental principles of data privacy and security, this research avenue will focus on the development of methodologies, the expansion of data sources, and the synthesis of privacy-preserving techniques. Our research roadmap is prepared to expand its influence beyond the prognosis of cardiac disease with a forward-looking approach. A variety of critical illnesses, including Parkinson’s disease, diabetes, liver cancer, skin cancer, and breast cancer, are all covered by the upcoming trajectory’s rehabilitation and therapy paradigms. We hope to use this multidimensional project to directly promote medical knowledge by utilising intelligent frameworks to enhance patient care in a variety of important health sectors.

## Figures and Tables

**Table 1:** Comparison of the accuracy obtained by different models corresponding to different communication rounds.

Communication Rounds	Models			
	**GA-SVM**	**SVM**	**Fed-SVM**	**Proposed**
100	58.1106	55.4501	54.9512	60.9323
200	74.8933	71.8891	75.9001	85.8879
300	90.0010	87.6431	90.7001	95.6734
400	92.0141	88.3317	91.7810	96.7812
500	93.6111	90.5351	93.9372	97.9441
600	94.7801	91.6670	94.6711	98.5780
700	95.6616	92.1332	95.1470	99.2016
800	96.8900	93.8824	96.7816	99.4888
900	97.9951	94.4672	97.4777	99.6721
1000	98.8771	95.8689	98.6712	**99.8582**

**Table 2:** Comparison of the accuracy obtained using different models corresponding to different communication volumes (in gigabytes).

Communication Volume (in Gigabytes)	Models			
	**GA-SVM**	**SVM**	**Fed-SVM**	**Proposed**
0.5	48.0122	35.4503	54.9550	60.9210
1.0	64.8910	57.8789	75.8991	85.9111
1.5	70.0000	67.6412	90.6753	95.7001
2.0	82.1462	78.3336	91.7825	96.8124
2.5	91.6186	80.5441	93.9409	97.9508
3.0	92.8201	91.6663	94.6720	98.7109
3.5	93.6609	92.1315	95.1333	99.1336
4.0	94.8900	93.8870	96.7898	99.4813
4.5	95.9951	94.4672	97.4671	99.6739
5.0	96.8720	95.8728	98.6733	**99.8972**

**Table 3:** Statistical summary of the total utility incurred by different models corresponding to different computation resource cost.

Computational Resource Cost	Models			
	**GA-SVM**	**SVM**	**Fed-SVM**	**Proposed**
1	5.0010	5.4506	15.9513	42.9505
2	7.1006	7.8913	19.8862	59.8991
3	10.1141	12.6421	22.6771	65.7767
4	14.2011	18.3331	30.7888	78.7870
5	17.2112	19.5401	38.9332	82.9405
6	19.0233	23.6561	40.6661	92.6703
7	21.6653	26.1460	45.1333	99.1311
8	22.8925	27.8887	48.7802	105.4812
9	25.9895	30.4751	55.4870	112.6710
10	28.8763	35.8720	65.6725	**120.8072**

**Table 4:** Statistical summary of the total utility incurred by different models corresponding to transmission cost.

Transmission Cost	Models			
	**GA-SVM**	**SVM**	**Fed-SVM**	**Proposed**
1.0	15.4459	10.0013	22.9504	52.9492
1.5	17.8920	12.1101	27.8889	60.0133
2.0	19.6433	14.1336	32.6666	66.6701
2.5	23.3313	16.0768	37.7779	73.7881
3.0	31.5400	18.0644	42.9421	75.9441
3.5	33.6702	20.1233	47.6567	77.6721
4.0	36.1333	23.6667	52.1280	79.1317
4.5	40.8867	27.8993	58.7801	83.4876
5.0	43.4670	34.9950	63.4669	87.6777
5.5	45.8720	39.8763	69.6770	**95.9111**

## Data Availability

Data will be made available on request from the authors.
